# Knowledge, attitude and practices related to diabetes among community members in four provinces in Kenya: a cross-sectional study

**Published:** 2010-10-06

**Authors:** Maina William Kiberenge, Zachary Muriuki Ndegwa, Eva Wangechi Njenga, Eva Wangui Muchemi

**Affiliations:** 1 Ministry of Public Health and Sanitation- Kenya; 2 National Diabetes Control- Kenya; 3 Diabetes Endocrinology Center- Nairobi; 4 Kenya Diabetes Management and Information Centre (DMI)

**Keywords:** Diabetes, knowledge, attitude, practices, community, Kenya

## Abstract

**Background:**

This cross-sectional study sought to establish the level of knowledge of diabetes among community members in rural and urban setups in Kenya and determine how this impacts on their attitude and practices towards diabetes.

**Methods:**

A face-to-face interview was done for selected respondents using a structured questionnaire for data collection.

**Conclusion:**

1982 respondents, 1151 (58.1%) female and 831 (41.9%) males aged between 13 and 65 years were interviewed. 539 (27.2%) of all the respondents had good knowledge of diabetes; of these 52% had tertiary education; 25% had secondary education while 14% and 9% had primary and no education, respectively. Only 971(49%) of the respondents had a positive attitude towards diabetes while 813 (41%) demonstrated good practices towards diabetes.

**Conclusion:**

This study indicates that the level of knowledge of diabetes in all regions in the country is very poor. It also indicates very poor attitudes and practices of the community towards diabetes. A comprehensive nationwide diabetes education programme is necessary to improve this situation.

## Background

The International Diabetes Federation estimated the prevalence of diabetes in Kenya to be about 3.3% in 2007 [1]. However, local studies have shown prevalence of 4.2% in the general population with a prevalence rate of 2.2% in the rural areas and as high as 12.2% in urban areas. The prevalence of impaired glucose tolerance is equally high 8.6% in the rural population, and 13.2% in the urban population [2].

Urbanization with adoption of “western lifestyles” has been incriminated in the abandonment of the healthier “traditional lifestyles” by people in developing countries. The traditional lifestyle was characterized by regular and vigorous physical activity accompanied by subsistence on high fiber, whole grain-based diet rich in vegetables and fruits [2,3]. Urban or even “western lifestyles” in rural areas have resulted in overreliance on motorized transport and consumption unhealthy diets rich in carbohydrates, fats, sugars and salts [4].

These lifestyles have contributed to a rise in levels of obesity and overweight in the population increasing the risk for diabetes. For instance, the 2003 Kenya Demographic and Health Survey about 20% of women and 7% of men in the country were overweight or obese [5]. Recent studies have shown even higher figure of 60.3% and 19.5% for women and men respectively in urban areas as compared to 22.6% and 10% in women and men respectively in rural areas [6].

The rise of these determinants of chronic diseases reflects the major forces driving social, economic and cultural change in the Kenyan society. These same factors are driving the epidemiological landscape with chronic non-communicable diseases becoming major contributors to the national disease burden [3].

Diabetes is now emerging as an epidemic of the 21st Century. It threatens to overwhelm the health care system in the near future [7]. Sadly, the majority of the people with diabetes in developing countries are within the productive age range of 45 to 64 years [3]. These are the same individuals who are expected to drive the economic engines of these countries in order to achieve the agreed international development goals. Besides their reduced productivity, diabetes further imposes a high economic burden in terms of health care expenditure, lost productivity and foregone economic growth [3].

To curb this scourge of diabetes, public health interventions are required to prevent diabetes or delay the onset of its complications. This will entail intensive lifestyle modification for those at risk of diabetes and aggressive treatment for those with the disease [8]. A high risk approach targeting individual at risk of diabetes and a population or public health approach aimed at reducing the risk factors for diabetes at the community are necessary.

Knowledge is the greatest weapon in the fight against diabetes mellitus. Information can help people assess their risk of diabetes, motivate them to seek proper treatment and care, and inspire them to take charge of their disease [9]. It is therefore in the interest of the country to design and develop a comprehensive health promotion strategy for diabetes mellitus and its related risk factors. It is equally important to design and implement suitable diagnostic, management and treatment protocols for people with diabetes.

This study therefore was conducted to assess the level of community awareness of diabetes and how this knowledge influences their attitude and practices in prevention and control of the disease. The findings will help in identifying population knowledge gap and their behaviour towards diabetes which will guide the development of prevention programmes in the country.

## Methods

This was a descriptive cross-sectional study involving 2000 people drawn from 8 districts in 4 provinces. The 4 provinces were selected from a total of 8 due to their high burden of diabetes as reported in the health management and information systems in the Ministry of Health. 2000 respondents were considered adequate as similar studies done in the country have worked with nearly equal number. The 4 provinces had a total of 23 districts, the districts were stratified into rural and urban districts based on their geographical location. Two districts, one rural and one urban were randomly selected from each province. Each of the 8 districts was assigned 500 respondents. The respondents were aged between 13 and 65 years. Only one respondent was interviewed for every household visited.

A medium sized four part questionnaire was designed by the researchers. It was peer reviewed by 5 colleagues including a biostatistician for validation of the questions. The questionnaire was then piloted on 10 respondents in Kajiado district which is a rural district next to Nairobi. This was done in order to assess the suitability of the contents, clarity, sequence and flow of the questionnaire. The questionnaire was then refined for final use. All questionnaires were in the English language, which is the national official language.

The first part of the questionnaire covered the respondent’s demographic information which included: name, age, sex, level of education, occupation and average monthly income.

Part two covered knowledge about diabetes. Knowledge on causes of diabetes was based on responses to a question on what they knew was the cause of diabetes. The options given were: lack of insulin, failure of the body to use insulin and consumption of lots of sugar or don’t know. for knowledge about signs and symptoms of diabetes, five options were given: frequent urination, excessive thirst, excessive hunger, weight loss, and high blood sugar. Knowledge of complications of diabetes was assessed by asking respondents to describe complications of the disease they knew. Options listed included, loss of vision, kidney failure, heart failure and stroke, poor healing wound and amputation. Respondents’ knowledge of diabetes was categorized as either good or poor depending on their responses to the knowledge areas assessed.

Part three of the questionnaire assessed the attitude of the respondents towards lifestyle characteristics such as diet, physical activity and health seeking behaviour.

Part four assessed what the respondents practiced in terms of adopting healthy lifestyles that promote diabetes prevention. This section looked at consumption of healthy diet, regular physical activity, avoidance of alcohol and tobacco use and regular medical checkup.

The questionnaire was administered by interviewers who were people with medical background knowledge of diabetes and included nurses, clinical officers and nutritionists. Before going to the field, the interviewers were taken through a one day training to acquaint themselves with the data collection tools and also to understand the whole concept. The interviewers then embarked on data collection by moving from house to house within their allocated areas. The first person to be encountered in the household meeting the age criteria was interviewed. For those who declined, a second person was interviewed and in their absence the next household was visited.

All filled questionnaires were then submitted to the survey supervisors who checked their completeness before the interviewer left that area. Where information was missing the interviewer revisited the respondent for further information unless they had initially declined to disclose. Upon processing of all the field data, analysis was done under the domain of descriptive statistics using SPSS software.

## Results

Of the targeted 2000 respondents, 1982 (99.1%) were interviewed in this study. There were more females 1151 (58.1%) than males 831 (41.9%) interviewed. 358 (18%) of the respondents had tertiary education, 737 (37.2%) had secondary education, 725 (36.6%) had primary education while 162 (8.2%) had no education at all.

Only 575 (29%) of respondents had good knowledge of signs and symptoms of diabetes while 1407(71%) of respondents had poor knowledge on what diabetes is. 518 (26.1%) could correctly identify the probable causes of diabetes mellitus while 1464(73.9%) could not. Only 523(26.4%) of the respondents could identify complications of diabetes they knew while 1459(73.4%) had very little or no knowledge of complications of diabetes (Table 1).

Overall on average 539(27.2%) respondents had good knowledge of diabetes while 1443 (72.8%) had poor knowledge of the disease. There was therefore no significant difference in knowledge levels between genders. The proportion of females who had good knowledge was 26.8% compared to 27.7% in males.

**Regional differences in level of knowledge**

Results revealed a significant is a disparity in the level of knowledge in different regions. Coast province had the lowest knowledge level of diabetes 118 (23.7%) followed by Nairobi 127 (25.5%), Eastern 140(28.9%) and Central 154 (30.8%), respectively. Nearly over 70% of all respondents from each of the four regions had poor knowledge of diabetes (Table 2).

**Variation of knowledge of diabetes with level of education**

**Figure 1:**
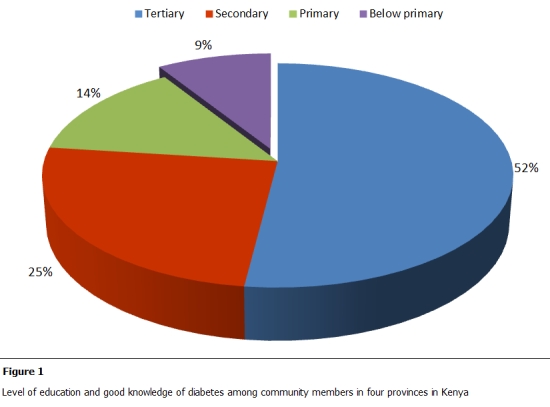
Level of education and good knowledge of diabetes

All the respondents with good knowledge were analyzed according to level of education. A direct relationship between level of education and good knowledge of diabetes was demonstrated. 52% of those who had good knowledge had tertiary education, 25% had secondary education, and 14% had primary education while 9% had no formal education (Figure 1).

**Community attitude and practices towards diabetes**

To assess the attitude of community towards diabetes, the attitude of people towards lifestyle characteristics such as diet, physical activity and health seeking behavior was assessed. Only 28% of respondents agreed with statements relating to willingness to engage in physical activity, changing eating habits and maintaining “good” body weights. A significant 813 (41%), of the respondents did not indicate any willingness to adopt these healthier lifestyles. 41% of all respondents had good practices while the rest 59% had bad practices in relation to diabetes prevention. 75% of the people interviewed had poor dietary practices, 72% did not participate in regular exercise and over 80% did not monitor their body weights.

**Relationship between practices and knowledge**

Further analysis of the relationship between community knowledge and practices provided valuable insights in the assessment of community attitude. 50.7% of people with good knowledge of diabetes had good practices as compared to 37.4% of people with poor knowledge of diabetes had good practices. Conversely, 49.3% of those with good knowledge had bad practices compared to 62.6% of those without knowledge (Table 3).

## Discussion

Most studies on the knowledge, attitude and practices of diabetes done in Africa and elsewhere target patients with diabetes. Unlike these, this study targeted the general population. We therefore lack adequate comparative data for community and our discussions are based on knowledge, attitude and practices of people with diabetes who in most cases have better exposure to diabetes education.

The findings of this study reveal a serious deficiency in knowledge of diabetes among community members in Kenya. Only 27.2% of the people interviewed had good knowledge of diabetes. Puepet et al., found a similar level of knowledge of diabetes, 30.2%, among patients with diabetes in Jos State, Nigeria [10]. Dinesh et al., in a study in western Nepal, noted a lack of awareness of diabetes even in patients who had had the disease for a long time [11]. Even in a developed country set up, Baradaran and Jones also found that knowledge about diabetes amongst ethnic groups in Glasgow was very low [12].

These findings underscore very important aspects of education to the community as far as diabetes is concerned. Firstly there is historical deficiency in knowledge about diabetes and inequalities in the quality of education reaching each region in the country. Similar findings were documented by Hawthorne and Tomlinson regarding Pakistani Moslems attending the Manchester Diabetic Centre [8,13]. Secondly the low level of community knowledge of diabetes reflects on the extent of health promotion for most chronic non-communicable diseases. At the moment, there are no comprehensive primary care programmes for diabetes in the country and diabetes health education is done within health facilities through microteaching and only targets those with diabetes. This therefore leaves the rest the public ignorant of the disease. Most of the diabetes health promotion efforts by different stakeholders are uncoordinated and the messages are not standardized due to lack of clear guidelines regarding diabetes education [12]. Lastly, there is even low knowledge of diabetes among health care workers who are expected to deliver health education to the community [14,15].

Community knowledge, culture and beliefs about diabetes is a prerequisite for individuals and communities to take action to control the disease. This knowledge affects their attitude and uptake of health services, including health education [12]. Yet research into health knowledge and beliefs around diabetes causation and prevention among the general community in Kenya is lacking.

Diabetes prevention interventions need to target health education directed to the community and the health care providers. Good knowledge of diabetes amongst care givers is directly related to the quality of care given by such providers. Education of patients, likewise, improves compliance to treatments and leads to favorable treatment outcomes. This is due to the direct influence of knowledge on the attitude and practices of both the care giver and the patients [16].

Over 49.3 % of those with good knowledge had poor practices as far as diabetes is concerned. Low knowledge of diabetes in the community may result in poor attitude however; this does not explain the poor practices even in people with good knowledge of the disease. Altamimi and Peterson demonstrated that women continued to consume sweetened foods, even though they knew about the deleterious impact of sugar on oral and dental tissues [17]. Knowledge does not always result in behavior change and need to be reinforced [18].

Since the knowledge referred to in this study was the conventional form obtained from the formal information, communication and education systems, the reason for good practice among 37.4% of people with no knowledge was associated with their indigenous knowledge. It is therefore important to identify interventions that reinforce peoples’ attitudes despite their levels of knowledge of a particular subject [19]. Proper education and awareness programs have previously been shown to change the attitude of the public regarding diabetes. Improving knowledge of the people can improve their attitude towards diabetes and in the long run change their practices to embrace healthier lifestyles such as eating healthy foods, and engaging in physical activity [20]. Such practices will minimize the risks for diabetes in the general public and delay the onset of complications in those already diabetic.

There is need for further in-depth studies to investigate the social cultural beliefs of health in Kenyan communities. These perceptions have reinforced unhealthy dietary habits even though people are aware of the relationship between these practices and chronic diseases such as diabetes [21].

There was marked regional discrepancies in the level of knowledge with Central province having relatively higher level of 30.8% and Coast province having the lowest at 23.7%. The differences in the level of knowledge or the low levels do not imply in any way that there is deficiency in intelligence in the various groups and communities in the different regions. It only implies a lack of exposure to knowledge about diabetes due to poor health education, inaccessibility of good health care services and also low literacy levels in some areas. This has previously been noted among patients with diabetes in a primary health care setting in South Africa [9] and among Pakistani Moslems with type 2 diabetes in Manchester [13].

Preventing disease potentially avoids and certainly postpones suffering and may have many other benefits that are difficult to quantify (e.g. impact on families), which may make it preferable to treatment. This study forms a baseline for the national diabetes awareness campaigns and demonstrates the wide knowledge gap which requires a concerted effort by those involved in diabetes management and education. A systematic education curriculum for diabetes education is essential for all levels of health care, from the community to the highest referral level. The community health education interventions for diabetes need to take into account the disparity and uniqueness which exist between gender, age groups and regions.

**Study limitations**

This survey did not identify those with diabetes among the respondents. Such people would have higher knowledge due to the patient education provided at the clinic. The questionnaires were in English and their administration depended on the translation of interviewers for the respondents to understand. The responses depended on the memory and truthfulness of the respondents which was assumed to be reliable. The entry of responses into the questionnaire depended on the interviewers’ interpretation of the response and was subject to misrepresentation. This was however reduced due to training of interviewers and use of people with medical background.

In this study, we did not ask the community about their sources of health information. Knowledge of these sources of information would have been useful in identifying the appropriate media for delivery of health promotion interventions. There is therefore need for further community surveys to identify sources of health information and the validity of the information delivered through such media.

## Conclusion

Knowledge about diabetes mellitus is a prerequisite for individuals and communities to take action to control the disease. However, research to assess knowledge deficiencies and their relation to health-seeking behavior is lacking in most developing countries. Diabetes education, with consequent improvements in knowledge, attitudes and skills, will lead to better control of the disease, and is widely accepted to be an integral part of comprehensive diabetes care.

## Competing interests

The authors declare no conflict of interest.

## Authors’ contribution

MWK participated in obtaining the ethical approval, study design, data analysis and in drafting the manuscript. NZ participated in study design, supervision of data collection and literature review. NE participated in the review of the manuscript and ME participated in review of the data and the manuscript.

## Acknowledgements

The authors would like to acknowledge the World Diabetes Foundation (WDF) for their financial support to carry out this study. We particularly appreciate the contribution of Scholastica Mwende, Onesmus Mwaura and Edward Ndungu for assisting in supervising the data collectors. We also appreciate Mr. Benson Maina and Retasi Strategic Solutions for assisting us in data entry and analysis. We appreciate the contribution of Dr. Kathreen Karekezi in the peer review of the manuscript.

## Figures and Tables

**Table 1:** Levels of community knowledge on different aspects of diabetes

**Knowledge of diabetes**	**Signs and symptoms**	**Causes**	**Complications**
Good knowledge	575 (2 9%)	518 (2 6.1%)	523 (2 6.4%)
Little or none	1407 (71%)	1464 (73.9%)	1459 (73.4%)
Total	1982 (100%)	1982 (100%)	1982 (100%)

**Table 2:** Regional differences in level of knowledge

		**Community Knowledge**	
**Province of residence**	**Good**	**Poor**	**Total**
Nairobi	127 (25.5%)	372 (74.5%)	499 100.0%
Coast	118 (23.7%)	380 (76.3%)	498 (100.0%)
Eastern	140 (28.9%)	345 (71.1%)	485 (100.0%)
Centra	154 (30.8%)	346 (69.2%)	500 (100.0%)
Total	539 (27.2%)	1443 (72.8%)	1982 (100%)

**Table 3:** Relationship between community knowledge and practices

			**Community practices**	
**Community Knowledge Level**	**High**	273 (50.7%)	266 (49.3%)	539 (100.0%)
	**Poor**	540 (37.4%)	903 (62.6%)	1443 (100.0%)
**Total**		813 (41%)	1169 (59%)	1982 (100.0%)
